# Human-animal bonds: first cross-sectional study in laboratory animal care professionals in Argentina

**DOI:** 10.3389/fvets.2026.1738072

**Published:** 2026-03-06

**Authors:** Gisela Ariana Marcoppido, Juan Santiago Guidobono, Marcos Trangoni, María Aluminé Mattana, Agustina Resasco, Silvina Laura Diaz

**Affiliations:** 1 Instituto de Patobiología/Instituto de Patobiologia Veterinaria (IP/IPVET), UEDD INTA-CONICET, Instituto Nacional de Tecnología Agropecuaria (INTA), Hurlingham, Argentina; 2 Consejo Nacional de Investigaciones Científicas y Tecnológicas, CONICET, Buenos Aires, Argentina; 3 Instituto de Ecología, Genética y Evolución de Buenos Aires (IEGEBA), CONICET, Universidad de Buenos Aires, Buenos Aires, Argentina; 4 Instituto de Biotecnología/Instituto de Agrobiotecnología y Biología Molecular (IB/IABIMO), UEDD INTA-CONICET, CICVyA, CNIA, INTA Castelar, Castelar, Argentina; 5 Facultad de Psicología, Universidad Nacional de Córdoba, Córdoba, Argentina; 6 Biological Research Facility, The Francis Crick Institute, London, United Kingdom; 7 Instituto de Biociencias, Biotecnología y Biología Traslacional, Facultad de Ciencias Exactas y Naturales, Universidad de Buenos Aires, Buenos Aires, Argentina; 8 Cátedra de Técnicas de Bioterio, Facultad de Farmacia y Bioquímica, Universidad de Buenos Aires, Buenos Aires, Argentina

**Keywords:** burnout (BO), compassion fatigue (CF), compassion satisfaction (CS), environmental enrichment, euthanasia, human-animal interactions, professional quality of life

## Abstract

Working with laboratory animals can bring satisfaction but may also result in workplace stress. The established bonds between laboratory animals and their caretakers can make a person feel physically, mentally or emotionally uncomfortable. This phenomenon has been described as Compassion Fatigue (CF), a combination of burnout (BO) and secondary traumatic stress (STS). Although CF has been recognized in the field of laboratory animal science, the information about laboratory animal workers is still scarce. Our aim was to investigate the prevalence of CF and Compassion satisfaction (CS) and identify risk factors in laboratory animal care professionals (LACP) in Argentina. We conducted our study during 2.024. A total of 106 LACP completed an online survey regarding social support, euthanasia, environmental enrichment, stress and pain caused to animals, professional quality of life and human-animal interactions. Components of CF were associated with less control over euthanasia procedures and the frequency of conducting the procedure, the use of toys as enrichment tools, and talking to animals and introducing themselves to society as LACP. CS was associated with the desire to add more enrichment and pride in communicating their jobs to others. These results provide empirical information that can contribute to the development of intervention policies to improve the quality of life of both the people who work with laboratory animals and the animals they care for.

## Introduction

In 1992, the nurse Carla Joinson introduced the term Compassion fatigue (CF) to describe a unique form of burnout suffered by nurses working in the emergency department. This workplace-associated stress was soon extended to workers in the human healthcare field (1). Later, Charles Figley equated CF to secondary traumatic stress, reinforcing the concept that this psychological syndrome represents the natural behaviors and emotions resulting from knowing about a traumatizing event experienced or suffered by a person and referred to CF as the “cost of caring” (2, 3). Therefore, two elements may be involved when experiencing CF: (a) the burnout (BO) characterized by feelings of exhaustion, unhappiness, disconnectedness, and insensitivity to the work environment; and (b) secondary traumatic stress (STS), explained by the feelings of being preoccupied with thoughts/experiences of individuals one has helped (4). The latter has a distinctive aspect involving invasive thoughts, avoidance, and associated fear, inducing more potent effects on people. In a positive pathway, workers in caring professions may also experience Compassion Satisfaction (CS), identified as the ability to perform their job well and contribute to the work setting and the greater good of society (5). Compassion Satisfaction, in contrast to CF, makes caregivers enjoy their work and engage more with the individuals they take care of, due to the strong bonds that may develop (4, 6).

In 2006 Figley initiated the extrapolation of existing nomenclature to caregivers in animal-related professions (7). The recognition of potentially vulnerability of laboratory animal care professionals to CF increased in animal-related professions and predisposing key factors were identified (4, 6, 8, 9). The dependency of laboratory animals on their caregivers and the time they share daily at work, help to forge strong bonds between animals and humans (10). In addition, there is frequently a personal cost to the caregiver who becomes emotionally attached to laboratory animals that may eventually be euthanized, experiencing moral ambivalence known as the caring-killing paradox (11, 12) and performing moral stress (13), presentations that lead to further reduction in the quality of life, associated with isolation, loss of empathy, substance abuse and feelings of anger, and sadness (8).

Several studies have analyzed the situation of laboratory animal care staff predominantly in the United States, Europe and Australasia, reporting higher levels of work-related stress, CF and BO than the general population (4, 14, 15). Besides characterizing this profession, these studies paved the way to develop support programs to apply in animal facilities. Although this activity/profession is disseminated worldwide, it may not be valid to extrapolate conclusions of these studies to other countries (16). Indeed, information collected at a local level is advantageous since cultural, legal, educational, and other conditions may affect differentially the prevalence and development of this psychologic entity.

Taking these factors into account, and since no analysis of the situation of laboratory animal caretakers in Argentina has been carried out, we undertook a cross-sectional study of human-animal relationships, which have an impact on both animal welfare and human welfare. We took advantage of the Professional Quality of Life Scale (ProQOL), developed for caring professions to measure how an employee feels in relation to their work as a helper (5). The ProQOL questionnaire is one of the most widely used instruments to measure the positive and negative aspects of caring for others, with good reliability and construct validity, and determines the prevalence of CF, discriminated in BO and STS, and CS in participants (5). We looked for risk or protective factors to develop CF to provide technicians with mental health support, build personal resilience as well as have elements to design, at long term, preventive/support programs of workplace stress.

## Materials and methods

### Ethical approval

The survey and informed consent protocols were approved by the Comite de Etica de la Investigacion (CEI), from the Sociedad Argentina de Investigaciones Clinicas, protocol No. 10883 (2024). All participants gave their voluntary informed consent attached to the questionnaire.

### Participants and procedures

The study was conducted in Argentina. The survey, presented in Google forms, was first sent in July 2024 to email addresses of institutions enrolled in the “Sistema Nacional de Bioterios” (SNB), a national record of animal facilities set by the National Ministry of Science, to which establishments adhere voluntarily. A remainder was sent in August 2024. The information was also disseminated by email or WhatsApp to personal contacts of the authors to increase the population/sample of respondents. The form closed in October 2024. All participants received a voluntary informed consent prior to completing the survey.

Participants over 18 years old were included, formerly or currently working with laboratory animals. We covered all experimental animals under a scientific protocol: domestic or captive wild species, terrestrial or aquatic animals, farm animals or classical species (mainly rodents).

All the questions were optional as requested by the CEI.

### Questionnaire

This survey was based on the study conducted by Dr. Megan LaFollette (4) with her written permission. Spanish translation of the entire survey was made by bilingual researchers (see Supplementary material). The original and the translated survey were approved by the CEI, with minimal modifications that were agreed after mutual consensus to adapt to the Argentinian idiosyncrasy.

### Demographic and work factors

The study was conducted in Argentina. Demographics included questions about gender, age, and highest level of education (Questions 1–3) (4).

Work factors included role, type of institution, primary type of research activity, main animal type they work with, years and hours per week working with laboratory animals (Questions 4–9). The work with laboratory animals included all types of activities, from changing cages to management roles.

### Animal stress, social support, enrichment, euthanasia, and human-animal interactions

Participants were asked to self-assess the degree of stress or pain they cause to the animals they work with, using the categories based on the Institutional Animal Care and Use Committee (CICUAE) guidelines from the Instituto Nacional de Tecnologia Agropecuaria (INTA), provided in the survey with examples, through a direct link (Question 10).

Regarding social support, participants were asked how often they talk to others about their work with laboratory animals and how often they feel like they have someone to count on when they are dealing with a painful and/or stressful situation related to their work involving animals. Respondents were also asked about how they introduce themselves to others (Questions 48–50). Enrichment practices were described in the survey as “the way in which the environment can be conditioned through novel techniques or elements so that animals can satisfy their physical, psychological and behavioral needs, in order to improve their daily comfort.” We surveyed participants about the control they have over the type and amount of enrichment provided, the possibility they have to increase the enrichment, and the frequency of use of different types of enrichment elements (Questions 11–13) (4).

Concerning euthanasia practices, we asked about the frequency, the methods employed, and the possibility to decide whether to carry on or not the procedure on the animals they care for (Questions 45–47) (4).

Human-animal interactions were evaluated by asking participants how strongly they agree or disagree with four actions: observe, pet, talk or name their laboratory animals (Questions 51–54) (4).

### Professional quality of life

The Professional Quality of Life (ProQOL) questionnaire assesses workplace stress and satisfaction to determine the prevalence of compassion fatigue, including burnout and secondary traumatic stress, as well as compassion satisfaction (5). It has good reliability and construct validity (10). Spanish translation of the ProQOL scale was made by bilingual researchers, and approved by the CEI, adapted to Latin America idiosyncrasy.

The ProQOL questionnaire asked 30 questions (Questions 14–43), measures two principal subscales: positive (CS) and negative (CF), this last one subdivided in two subscales of BO and STS (5).

### Data analysis

#### Variable coding

We only included the answers of participants who responded at least 50% of the questions per measure, regardless of whether or not they practiced euthanasia at least once in their professional lives. To assist with analysis, categorical response options that resulted in <5 responses collapsed into larger categories.

Types of laboratory animals were coded into larger categories. Rats and mice remained in their own categories whereas guinea pigs, hamsters, and rabbits were coded as “traditional laboratory animals.” Farm animals' category included bovines, sheep, camelids, horses, poultry, and goats. Invertebrates grouped crustaceous, insects and bivalves. Companion animals (cats and dogs), fish, and small wild mammals were collapsed as “others.”

### Quantitative analysis

Data analysis was conducted in two consecutive stages: an initial descriptive and qualitative analysis to characterize the study population and their work practices, followed by an inferential analysis to identify factors associated with Professional Quality of Life.

#### Descriptive and qualitative analysis

Demographic, work-related, and specific practice data (social support, euthanasia, environmental enrichment, and human-animal interactions) were summarized using descriptive statistics. For categorical variables, absolute frequencies and percentages were calculated, using the total number of valid responses for each question as the denominator, given their optional nature. For continuous variables (age, years of experience, weekly working hours), the mean, standard deviation (SD), and range were calculated. The euthanasia methods reported by participants were analyzed through a qualitative content analysis, where two of the authors independently coded the responses into predefined categories, resolving discrepancies by consensus.

#### Inferential analysis of ProQOL components

As a first step, BO, STS and CS scores were constructed using information obtained from the adapted ProQOL questionnaire (5). Of the 68 questions in the questionnaire, 10 were used to construct each score (30 in total across the 3 scores) as outlined in “The Concise ProQOL Manual: The Concise Manual for the Professional Quality of Life Scale, 2nd Edition” (5). For the calculation of each score, only the responses of participants who answered all 10 questions associated with its construction were considered. The remaining 38 questions in the questionnaire were used as explanatory variables. As a next step, three databases were designed containing one of the three calculated scores and the explanatory variables taken from the questionnaire. As a final step, missing data were searched for in each of the three databases, eliminating participants who had any questions unanswered. For this reason, each database contained a different number of observations.

Once the database design process was completed, the collected information was statistically analyzed. For this purpose, the statistical program R (17) was used through the integrated development environment RStudio (18). Initially, evidence of collinearity between the explanatory variables was sought, performing a multiple correlation test using the “correlation” function in the “rcompanion” package (19). During this process, only the 12 mutually independent variables of the 38 original variables were retained. Next, we created saturated models (general linear models using the “lm” function of the R statistical program) (17) containing the retained explanatory variables and each of the estimated scores. Using each of the saturated models as input, three step-wise analyses were performed, one for each score, using a two-way selection algorithm (using the “step” function of the R statistical program) (17), which adds or removes an explanatory variable, improving model likelihood but penalizing complexity. This process is repeated until no modification improves the model. Once the final model was obtained, and after evaluating the assumptions of normality and homogeneity of variance, Type II ANOVA tables were performed (this type of ANOVA measures the individual contribution of each variable to the model, controlling for the others, and is appropriate when there are no interactions, as in this case, between the factors) to observe the significance of the variables retained by the step-wise process. Finally, Tukey's *post-hoc* tests were performed (using the “emmeans” function in the homonymous package) (20) to examine the pairwise differences between the different levels of the explanatory variables that showed significant effects.

The model assumptions and ANOVA type II tables were evaluated with the “car” package (21) and the graphs that expose the results obtained were made with the “ggefects” (22) and “ggplot2” (23) libraries.

## Results

### Demographics and work type

A total of 106 individuals completed the survey. Information concerning demographic aspects and work type is presented in Table 1. Most respondents, around 70%, were females and the average age was 39.6 years-old, with 82.1% of participants equal or under 50 years. On average, participants had worked with laboratory animals for 11.3 years and spend 15.7 h per week in their work.

**Table 1:** Demographic and work information (*N* = 106).

**Variable**		***N* (%)**
Gender	Female	69 (65.7%)
	Male	35 (33.3%)
	Not answered	1 (1%)
Education	High school degree	3 (2.8%)
	College degree	6 (5.7%)
	LATG degree	11 (10.4%)
	Bachelors	46 (43.4%)
	PhD	17 (20.5%)
	Postdoc	24 (28.9%)
Institution	University	31 (29.5%)
	Research Institution	67 (63.8%)
	Private lab	1 (0.95%)
	Government	6 (5.71%)
Research type	Basic	63 (59.4%)
	Applied	20 (18.9%)
	Animal production	23 (21.7%)
Animal species	Rats	18 (17%)
	Mice	63 (59.4%)
	Guinea pigs	2 (1.9%)
	Hamsters	1 (09%)
	Rabbits	1 (0.9%)
	Farm animals	11 (10.3%)
	Fishes	2 (1.9%)
	Invertebrates	5 (4.7%)
	Small wild mammals	2 (1.9%)
	Companion	1 (0.9%)
Role	LATG	19 (17.9%)
	Veterinarian	5 (4.7%)
	Researcher	52 (49%)
	Manager	6 (5.6%)
	LAT	10 (9.4%)
	Others	4 (3.7%)
**Continuous data**	**Mean** ±**SD**	**Range**
Age	39.6 ± 10.5	24–68
Years working with lab animals	11.3 ± 9.2	0–44
Hours/week working with animals	15.7 ± 13.3	1–40

Most of the participants (60%) worked in basic research with the rest equally distributed between applied research and animal production. Half of the respondents were Researchers, and around 20%, Laboratory Animal Technologists (LATG), a 3.5-year degree offered by the Universidad de Buenos Aires (UBA). Around 10% were Laboratory Animal Technicians (LAT), with Veterinarians only representing 5% of the participants.

Finally, the most common species reported were mice (59.4%), rats (17%), and farm animals (10.3%).

### Social support, animal stress, euthanasia, enrichment and human-animal interactions

**Figure 1:**
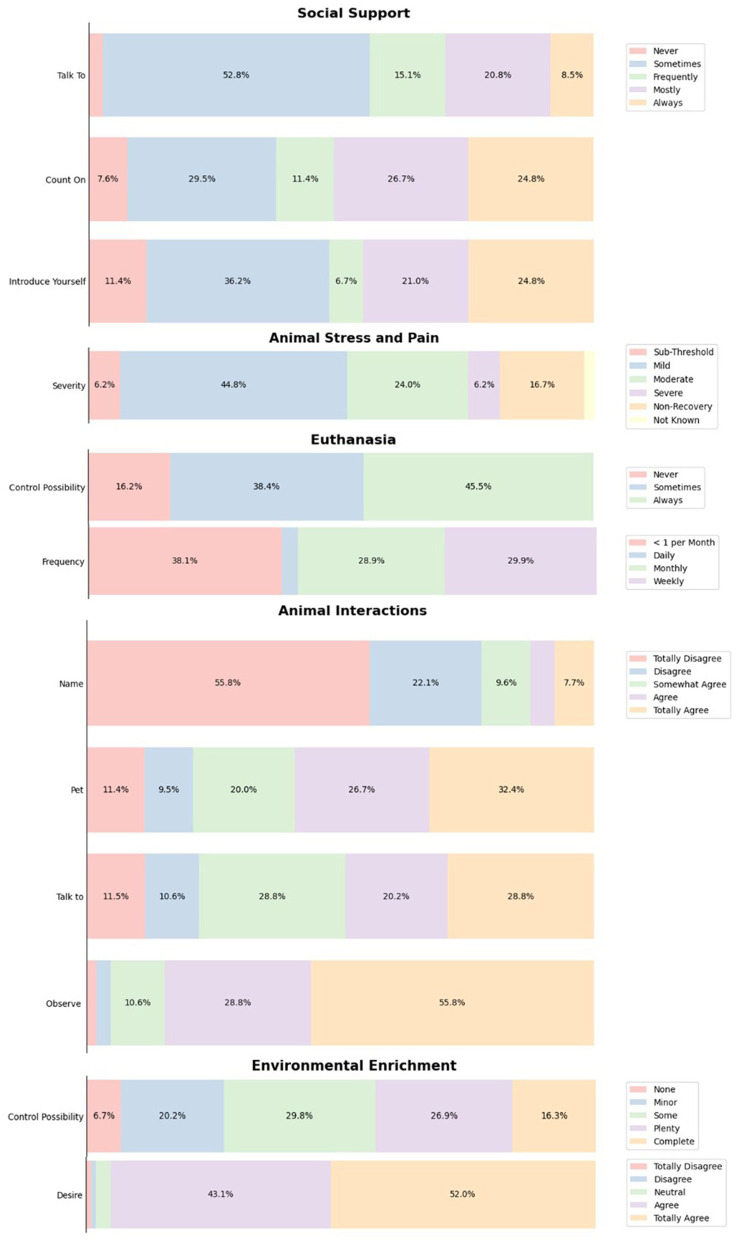
Descriptive statistics of several aspects related to laboratory animal personnel. Data shown represent the percentage of the different possible answers to the questions included in an online survey addressed to individuals working with laboratory animals in Argentina. Topics depicted covered social support, animal stress/pain, euthanasia, enrichment, and human-animal interactions.

Results about these aspects are presented in Figure 1. When asked about social support, most of the participants (102 out of 105) showed to have someone to talk on their organization from sometimes to always when they are dealing with stressful situations with laboratory animals, while 63.1% (65 out of 103), count with social support (family and friends) almost all the time.

Almost half of participants, 45.2%, introduce themselves as someone who works with laboratory animals from always to most of the time, while 43.4% of individuals do it from sometimes to frequently. Only 12 individuals (11.3%) never present themselves as laboratory animals worker.

Concerning animal stress and pain, 96 participants decided to answer the questions, of which 51% of participants reported to carry out minor to little painful procedures, while 23.9% performs moderate painful procedures. Only six individuals (6.25%) cause severe pain to the animals and 16 (16.6%) participants reported conducting non-recovery procedures. Surprisingly, two participants (2.08%) were not aware of the degree of pain they can cause with the procedures they perform.

In relation to euthanasia, 98 participants perform these procedures. Most of the participants (58.1%) perform euthanasia weekly or monthly, 37.6% individuals reported killing animals less than once per month while only three participants (3%) reported to do euthanasia daily. The remaining individuals decided not to answer about this procedure. Most of participants, 83.6%, reported some degree of control, from always to sometimes, over euthanasia protocols, while 16.3% do not have the possibility to decide over this procedure.

According to our survey, rats, mice and traditional laboratory animals (guinea pigs, hamsters and rabbits) and poultry are euthanatized using primarily inhalatory agents (CO_2_), while farm animals (bovines, sheep, camelids, and goats) are euthanized with chemical (barbiturates) and physical (stunning) procedures, followed immediately by exsanguination. In the case of invertebrates (crustaceous, insects, and bivalves) and fish, the participants reported decapitation and hypothermia as common methods. Companion animals (cats and dogs) and small wild mammals are usually killed by barbiturates overdose.

With respect to enrichment, most of the participants have some degree of control over techniques or elements: while 43.3% have plenty or complete control over enrichment, 50% have some or little control. Only seven participants declared not having control over enrichment at all. Despite this, 91.5% would like to provide more environmental enrichment to the animals they care and use.

Positive attitudes and promotion of human-animal relationships were observed as a result of our study. Almost all participants indicated that they observe their animals, while a high proportion indicated that they talk and pet the animals under their care. However, most of the respondents reported not naming animals.

### Professional quality of life

**Figure 2:**
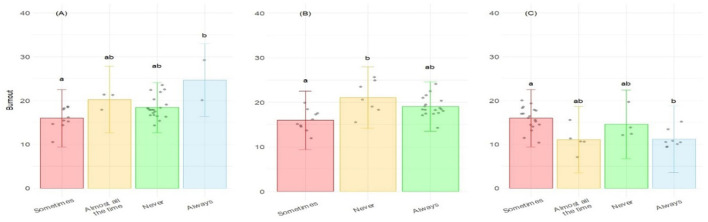
Level of Burnout corresponding to the ProQOL according to **(A)** the frequency of including toys as enrichment; **(B)** the incapacity to decide about euthanasia on the animals the individuals care for; and **(C)** the decision to introduce themselves to society as someone who works with laboratory animals. Bars represents the level of Burnout for each category of answers. Different letters correspond to differences statistically significant.

The PROQOL analysis was associated with several factors. According to the step-wise analysis, four variables were retained for Burnout, with three of them being statistically significant (Table 2). A higher BO was associated with the frequency of including toys as enrichment (*p* = 0.015), the possibility of deciding to practice the euthanasia procedures on the animals they care for (*p* = 0.020), and to introduce themselves to others as someone who works with laboratory animals (*p* = 0.016). *Post-hoc* analysis showed that, for the frequency of using toys as enrichment, the only significant differences were observed between “Sometimes” and “Always” categories, not being conclusive for the other pair-wise comparisons (Figure 2A). Concerning the possibility to decide on euthanasia procedures, the only significant differences were observed between “Sometimes” and “Never” categories (Figure 2B). Finally, for the way people introduce themselves to society, significant differences were observed only between “Sometimes” and “Always” categories (Figure 2C).

**Table 2:** ANOVA type II table of the statistical analysis on Burnout index.

**Variable**	**Sum.Sq**	**df**	***F*-value**	***p*-value**
Frequency of including toys	133.420	3	4.382	0.015
Frequency of conducting euthanasia	72.901	4	1.796	0.166
Euthanasia decision on animals	95.534	2	4.706	0.020
Introduce themselves as laboratory animals worker	129.652	3	4.258	0.016
Residuals	223.304	22		

Table entries correspond to: sum of squares (Sum. Sq), degree of freedom (df), Fisher's F-value (*F*-value) and estimated probability of type 1 error (*p*-value).

**Figure 3:**
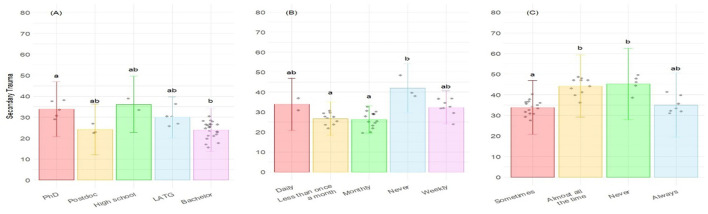
Level of Secondary traumatic stress corresponding to the ProQOL according to **(A)** the level of education; **(B)** the frequency of conducting euthanasia; and **(C)** the frequency of talking to animals as enrichment factor. Bars represent the level of STS for each category of answers. Different letters correspond to differences statistically significant.

For the other component of CF, the STS, five variables were retained (Table 3), with three of them being statistically significant. A higher STS was associated with the level of education (*p* = 0.006), the frequency of talking to animals as enrichment factor (*p* = 0.003), and the frequency of conducting euthanasia on the animals they care for (*p* = 0.010). *Post-hoc* analysis showed that, for the level of Education, the only significant differences were observed between “PhD” and “Bachelor” categories, not being conclusive for the other pair-wise comparisons (Figure 3A). For the frequency of euthanasia, significant differences were detected between “Never” and both “Monthly” and “Less than once a month” categories (Figure 3B). Concerning the frequency of talking to animals as an enrichment tool, significant differences were observed between “Sometimes” and both “Almost all the time” and “Never” categories (Figure 3C).

**Table 3:** ANOVA type II table of the statistical analysis on Secondary traumatic stress index.

**Variable**	**Sum.Sq**	**df**	***F*-value**	***p*-value**
Level of education	483.740	4	4.921	0.006
Frequency of conducting euthanasia	432.300	4	4.397	0.010
Euthanasia decision on animals	106.870	2	2.174	0.140
Introduce themselves as laboratory animals worker	115.050	3	1.560	0.230
Frequency of talking to animals	468.770	3	6.358	0.003
Residuals	491.540	20		

Table entries correspond to: Sum of squares (Sum. Sq), Degree of freedom (df), Fisher's F-value (*F*-value) and estimated probability of type 1 error (*p*-value).

**Figure 4:**
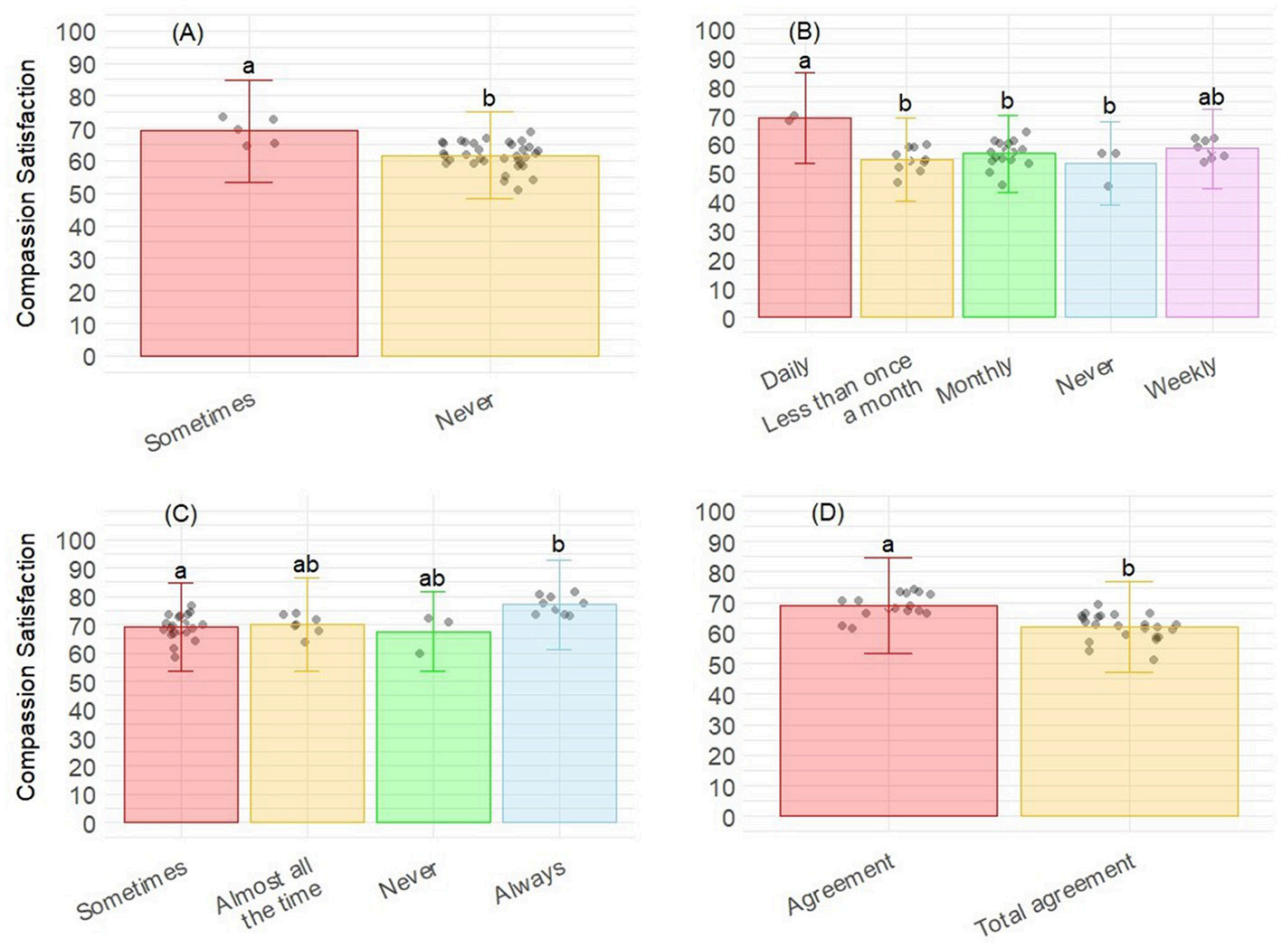
Level of Compassion satisfaction corresponding to the PROQOL according to **(A)** the frequency of putting hangers as enrichment; **(B)** the frequency of conducting euthanasia; **(C)** introduce themselves as someone who works with laboratory animals; and **(D)** the desire to add more enrichment. Bars represent the level of Compassion satisfaction for each category of answers. Different letters correspond to differences statistically significant.

**Figure 5:**
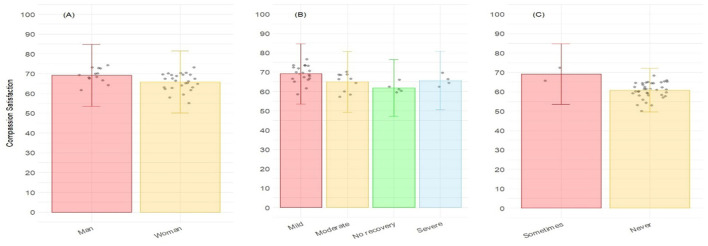
Level of Compassion satisfaction corresponding to the PROQOL according to **(A)** the gender; **(B)** the amount of stress caused to animals; and **(C)** the frequency of using music as enrichment. Bars represent the level of Compassion satisfaction for each category of answers.

Finally, seven variables were retained for Compassion Satisfaction (Table 4), being statistically significant four of them, and the rest showing a trend. Higher CS was associated with the desire to add more environmental enrichment (*p* = 0.003), introduce themselves as someone who works with laboratory animals (*p* = 0.010), the frequency of conducting euthanasia (*p* = 0.032), and the frequency of putting hangers as an enrichment tool (*p* = 0.018). *Post-hoc* analysis showed that, for the frequency of putting hangers as enrichment, significant differences were observed between “Sometimes” and “Never” categories (Figure 4A). For the frequency of euthanasia procedures, significant differences were observed between “Daily” and the three categories “Never,” “Monthly,” and “Less than once a month” (Figure 4B). Concerning the decision to introduce themselves as people working with laboratory animals, significant differences were observed between “Sometimes” and “Always” (Figure 4C). For the desire to include more enrichment, significant differences were observed between “Agreement” and “Total agreement” categories (Figure 4D). Finally, a trend to show significant differences was also detected for the gender (*p* = 0.090; Figure 5A), for the amount of stress caused to animals (*p* = 0.091; Figure 5B), and for the frequency of using music as enrichment (*p* = 0.094, Figure 5C). No *post-hoc* analysis was conducted for trends.

**Table 4:** ANOVA type II table of the statistical analysis on Compassion Satisfaction index.

**Variable**	**Sum.Sq**	**df**	***F*-value**	***p*-value**
Gender	80.770	1	3.133	0.089
Amount of stress caused to animals	186.680	3	2.413	0.091
Frequency of putting hangers	167.650	1	6.502	0.018
Frequency of using music	78.350	1	3.039	0.094
Frequency of conducting euthanasia	326.720	4	3.168	0.032
Introduce themselves as laboratory animals worker	368.240	3	4.761	0.010
Desire to add more environmental enrichment	288.810	1	11.201	0.003
Residuals	618.830	24		

Table entries correspond to: sum of squares (Sum. Sq), degree of freedom (df), Fisher's F-value (*F*-value) and estimated probability of type 1 error (*p*-value).

## Discussion

The human-animal interaction is an important factor in animal experimentation. In previous studies we analyzed information regarding welfare indicators in rodent facilities of our country (24), but up to our knowledge, we present herein the first cross-sectional study conducted in Argentina exploring the relationship between laboratory animals and their caregivers. Based on the report published by multiple authors (4, 6, 14, 25), we surveyed different aspects of working with laboratory animals like social support, the stress or pain caused to animals, the human-animal interactions, aspects linked to euthanasia procedures, and other potential risk factors that may influence the emotional profile of those working with laboratory animals. We surveyed 106 individuals performing different roles, working with multiple species, in different research topics and type of facilities.

Answers concerning social support showed some disparity which may reflect different work contexts. Particularly, people that “sometimes” introduce themselves to society as animal care workers showed a significant higher risk to develop BO compared to people that “always” reveal their job when introducing themselves. These results could be associated with the societal stigmatization suffered by laboratory animal professionals (16) as well as moral stress (13) which may also lead to more social isolation (4). These results could match with other reports that consider a sensitive issue working with laboratory rodents (6, 25). In turn, people that “always” introduce themselves as animal caregivers have also shown a significantly higher level of CS compared to the workers who “sometimes” introduce themselves as working with laboratory animals. These results reveal complementary aspects of individual attitudes, possibly indicating that the capacity to put our work in words may reflect how proud we are of the job we carry on and, moreover, this attitude may influence our emotional states. Remarkably a low proportion of participants are comfortable talking about their work from “frequently” to “always,” but this variable was not statistically related to any component of CF. It is interesting to mention that Argentina is an agricultural country where people is, in a certain way, habituated to listen about animals, and therefore there may be fewer attitudes of rejection, in general, than in more industrialized countries.

Being aware of the suffering we can cause to animals is a principal responsibility of animal users. It is thus unexpected to have two participants that did not know about the stress or pain they cause to animals, because these animals would not receive any caring/healing measure in case they need it. In addition, around 7% of respondents reported to carry out severe procedures. This appears to be in line with European data that show 9%−10% of severe procedures conducted in the last 5 years for which Statistics are available (European Commission, Summary Report on the statistics on the use of animals for scientific purposes in the Member States of the European Union and Norway in 2022) and around 5% in UK (Annual statistics of scientific procedures on living animals, Great Britain 2024, Home Office, 23-10-2025). It is important to mention that Argentina lacks a national law protecting animals used for experimental or educational purposes, although several attempts has been done by the LAS community for more than 40 years (24). In this sense, not only the laboratory animals but also the people in charge of these animals are not protected by a specific law. The main reasons for not having a Law, when other countries in the region, like Brazil and Uruguay, have its own Legislation is the lack of political will to discuss a topic so contradictory in a society, that progressively has a more positive and protective view over animals (26).

Most of the respondents to our survey conduct euthanasia “monthly” or less frequently, and almost half of them reported to “always” have control over this procedure. As mentioned previously in the context of the caring-killing paradox, one of the hardest tasks for animal caregivers is euthanasia (27). This is in part reflected by the fact that aspects linked to euthanasia were found as explanatory variables for the three emotional entities in our work. People that “never” can decide on conducting euthanasia are at higher risk of developing BO than those who “sometimes” can decide it. Similarly, the study from LaFollette et al. (4) reported that the control on euthanasia procedures is an explanatory variable for both, BO and STS. Surprisingly, in our survey, people that “never” perform euthanasia appeared to be at higher risk to develop STS, a counterintuitive result that may be explained because only 3 individuals answered “never” in this category. Indeed, this is the main limitation of this study: the low number of respondents retained for certain explanatory variables in each emotional state. This limitation also affects the interpretation of presenting satisfaction feelings (CS), because individuals that declared to “daily” perform euthanasia appear to have a higher probability to present CS compared to the other categories. Alternatively, we can hypothesize that in laboratory animals field, euthanasia is the expected necessary outcome of a project and the animal contribute to the research. The predictability and the execution of an experiment that includes the euthanasia of animals could be a mayor key factor to mitigate the negative emotional impact of the high frequency procedures (4). Indeed, healthcare workers showed that having control over difficult work situations has a significant direct relationship with perceived stress and this could happen in laboratory animal personnel (4). All in all, drawing conclusions from these results may be misleading and not conclusive, but certainly the possibility of being affected by this activity is high and this is thus an issue that deserves exhaustive research. In addition, it is interesting to remark that, although a slight proportion, some participants of the survey are LATG, it means laboratory animal technologists that have obtained a professional degree at University, a unique program that lasts 3 and a half years and prepare technicians with solid arguments (22). This academic frame prepare caregivers to deal with negative feelings and some of the discouraging activities that face this profession, like conducting euthanasia.

As in other study (25), the positive emotions that result from providing some kind of enrichment to animals may help to confront feelings of BO and STS. Although the use of certain elements as environmental enrichment, like toys, hangers, or talk to animals, were found as explanatory variables of the three emotional states, again it is difficult to evaluate the influence of these factors in our context, given the low quantity of participants and the fact that certain elements are species-specific, and therefore, not representative of all the animals. For example, we found that the use of hangers in experimental poultry was associated with CS as it was the case for the desire to improve enrichment. These results highlight the importance of increasing enrichment as a tool for improving the connection between animals and people, to level up the welfare of laboratory animals and to enhance the professional quality of life of laboratory personnel.

Results concerning human-animal interactions, shown that professionals that “always” observe, pet, and talk to animals were as many as those that have these attitudes “sometimes,” whereas a very small proportion of professionals indicated that they name their animals. We hypothesize that the distribution of these attitudes is related to the fact that most of the respondents worked with mice and rats (~60 and ~20%, respectively), which are species more difficult to individualize and thus, to establish a closer bond. Regarding these results, some studies established that there is a variability in the moral status that we attribute to some larger mammals (in this case associated with farm animals) compared to some smaller mammals (mice) and this moral concern is also present when research use companion animals and wild animals compare with food animals (28).

## Conclusion

This survey provides valuable information about laboratory animal personnel's professional quality of life in Argentina.

The results highlight the importance of considering the animal stress and pain inflicted to them, with continuing training and education sessions for laboratory animal personnel, the creation of social support groups inside the institution, which can develop interventions to decrease CF symptoms and increase CS performance. Enrichment should be seen as a close connection between animals and humans and a key factor for enhancing human-animal interactions, improving human wellbeing and animals' welfare. On the contrary, the high frequency and lack of control over euthanasia procedures may be detrimental for animal careworkers.

These results provide empirical information that can contribute to the development of intervention policies to improve the quality of life of both the people who work with laboratory animals and the animals they care for.

### Limitations

Even though in Argentina, the population of people who work with laboratory animals is small, a limitation of the study was the low response rate of personnel working with lab animals across the country. The communication strategy should be revised for further surveys and data collection.

## Data Availability

The original contributions presented in the study are included in the article/Supplementary material, further inquiries can be directed to the corresponding author.
