# Supplementary material for: Human-animal bonds: first cross-sectional study in laboratory animal care professionals in Argentina
Source: Front Vet Sci. 2026 Mar 6;13:1738072. doi: 10.3389/fvets.2026.1738072 (PMC13003596; doi:10.3389/fvets.2026.1738072)
Supplement: Supplementary file 1 [file Data_Sheet_1.pdf]

Bienestar en Animales de laboratorio-Bienestar en personal encargado del cuidado de animales de laboratorio

La siguiente encuesta está basada en un trabajo realizado por Megan LaFollette, et. al., (2020), en personal de animales de laboratorio de USA y Canadá (doi: 10.3389/fvets.2020.00114). La traducción y difusión de la misma, asi como la compilación de los resultados, está a cargo de las Dras. Gisela Ariana Marcoppido (INTA), Silvina Diaz (CONICET) y Agustina Resasco (The Francis Crick Institute). El objetivo de la misma es generar información empírica en Argentina, que pueda contribuir al desarrollo de políticas de intervención para mejorar la calidad de vida de las personas que trabajan con animales de laboratorio y de los propios animales que cuidan.

Esta encuesta está diseñada para ser contestada por personal que trabaja con animales de laboratorio en instituciones tanto públicas y como privadas. Se considerarán todas las especies domésticas o silvestres en cautiverio, con fines científicos, de cría de animales para futuros experimentos o educativos.

La encuesta está conformada por 55 preguntas, que demorará unos 30 minutos en completar. Su participación en esta encuesta es totalmente voluntaria, y todas las preguntas tienen la posibilidad de no ser respondidas. Además, su identidad e información personal serán tratadas como información confidencial, y protegidas mediante la anonimización (borramos su nombre)/codificación (usamos un código en lugar de su nombre) de sus datos, de manera que no se lo podrá identificar. Tampoco los compartiremos con otros investigadores. Si usted lo desea, al final de la encuesta puede anotarse de manera voluntaria para participar de una entrevista semi-estrucutrada via zoom (de forma anónima y confidencial), con el fin de contextualizar las experiencias vividas en su trabajo diario con los animales de investigación e identificar los puntos críticos, capacidades y actitudes necesarias para mejorar la calidad de vida tanto del personal como del animal.

Los resultados de este estudio se difundirán en congresos y reuniones científicas, y en lo posible se publicarán, en revistas científicas o en páginas de internet, en las cuales nunca podrán ser identificado. Si usted lo desea, puede aclarar al final de la encuesta si quiere recibir los resultados de lo mismo.

Demografía y factores asociados al trabajo

1. Edad (por favor, escriba únicamente el número en años). En caso de no querer responder, por favor escriba 'No deseo responder' o similar.

____

2. Género

Marca solo un óvalo.

- Hombre
- Mujer
- Hombre trans
- Mujer trans
- No binario
- No quiero contestar
- Otro: ____________________

3. Máximo nivel educativo alcanzado

Marca solo un óvalo.

- Primario
- Secundario
- Terciario/Técnico
- Técnico universitario en gestión integral de bioterios (UBA)
- Universitario de grado
- Doctorado
- Postdoctorado
- No quiero contestar

4. Cuál es su trabajo actual con animales de laboratorio?

Selecciona todos los que correspondan.

- [ ] Estudiante de grado
- [ ] Técnico de laboratorio
- [ ] Bioterista
- [ ] Estudiante de postgrado
- [ ] Encargado de bioterio
- [ ] Investigador postdoctoral
- [ ] Investigador principal
- [ ] Veterinario de animales de laboratorio
- [ ] Personal de apoyo
- [ ] Otro personal de investigación
- [ ] No quiero contestar
- [ ] Otro: ____________________

5. En qué tipo de institución desarrolla sus tareas?

Selecciona todos los que correspondan.

- [ ] Universidad
- [ ] Instituto de investigación
- [ ] Empresa privada
- [ ] ONG
- [ ] Organismo gubernamental
- [ ] No quiero contestar
- [ ] Otro: ____________________

6. Cuál es la actividad principal en la que usted está involucrado/a?

Marca solo un óvalo.

- ( ) Investigación básica
- ( ) Investigación aplicada
- ( ) Regulatoria
- ( ) Producción
- ( ) Educativa
- ( ) No quiero contestar
- ( ) Otro: ____________________

7. Cuántos años ha trabajado con animales de laboratorio? (por favor, escriba únicamente el número). En caso de no querer responder, por favor escriba 'No deseo responder' o similar.

____

8. Cuántas horas de contacto directo y/o indirecto dedica por semana al trabajo con animales? (por favor, escriba únicamente el número). En caso de no querer responder, por favor escriba 'No deseo responder' o similar.

____

9. Con qué tipo de animal usted trabaja en mayor proporción?

Marca solo un óvalo.

- Ratas
- Ratones
- Cobayos
- Hámsters
- Conejos
- Primates no humanos
- Cerdos
- Ovejas
- Bovinos
- Camélidos
- Equinos
- Aves de corral
- Peces
- Reptiles
- Anfibios
- Insectos
- Caninos
- Felinos
- No quiero constestar
- Otro: ____________________

Estado de los animales bajo vuestro cuidado

10. En general, cuánto estrés o dolor causa en los animales las investigaciones que usted conduce? Ver anexo I ***(link)***

Marca solo un óvalo.

- No recuperación
- Sub-umbral
- Leve
- Moderado
- Severo
- Desconozco
- No aplica
- No quiero contestar

11. Cuánto control tiene usted sobre el tipo y cantidad de enriquecimiento ambiental * que se usa con los animales con los que trabaja? *Consideramos como enriquecimiento ambiental a aquellas formas en que se puede acondicionar el ambiente, a través de Técnicas o elementos novedosos para que los animales puedan satisfacer las necesidades físicas, psicológicas y comportamentales, a fin de mejorar su confort diario

Marca solo un óvalo.

- Ninguno
- Poco
- Algo
- Mucho
- Completo
- No quiero contestar
- No aplica

12. Quisiera poder brindar más enriquecimiento ambiental a los animales con los que trabaja?

Marca solo un óvalo.

- Totalmente en desacuerdo
- En desacuerdo
- Indiferente
- De acuerdo
- Totalmente de acuerdo
- No quiero contestar
- No aplica

13. En los últimos años, cuan seguido utilizaron los siguientes enriquecimientos:

Marca solo un óvalo por fila.

	Nunca	A veces	La mitad del tiempo	Casi todo el tiempo	Siempre	Desconozco	No aplica	No quiero contestar
**Alojamiento colectivo**								
**Hablar con los animales**								
**Alimentos palatables (heno, fruta, verduras)**								
**Juguetes**								
**Alojamiento elevado**								
**Refugios escondidos**								
**Material para construcción de nido**								
**Areas de descanso**								
**Perchas**								
**Ejercitación**								
**Espejos**								
**Cambios de colores y texturas**								
**Música relajante o natural**								
**Radio**								
**Bateas con arena/tierra**								
**Material para roer**								

ProQol (Calidad de vida en el entorno profesional)

El trabajo que usted hace con los animales de laboratorio, puede ser dificultoso, ya que debe cuidarlos en la salud y en la enfermedad. Su interacción con sus animales tiene aspectos positivos y negativos. Queremos formularle preguntas en base a su experiencia, tanto positiva como negativa, en su trabajo actual. Seleccione cada respuesta reflejando honestamente con que frecuencia usted experimenta cada circunstancia en los últimos 30 días.

14. Soy feliz

Marca solo un óvalo.

- Nunca
- A veces
- Seguido
- Casi siempre
- Siempre
- No quiero contestar

15. Me genera preocupación /angustia atender animales bajo ciertos protocolos experimentales

Marca solo un óvalo.

- Nunca
- A veces
- Seguido
- Casi siempre
- Siempre
- No quiero contestar

16. Me da satisfacción cuidar/usar a los animales de laboratorio

Marca solo un óvalo.

- Nunca
- A veces
- Seguido
- Casi siempre
- Siempre
- No quiero contestar

17. Siento conexión con otras personas

Marca solo un óvalo.

- Nunca
- A veces
- Seguido
- Casi siempre
- Siempre
- No quiero contestar

18. Me asustan los ruidos inesperados

Marca solo un óvalo.

- Nunca
- A veces
- Seguido
- Casi siempre
- Siempre
- No quiero contestar

19. Me siento revitalizado después de trabajar con los animales que cuido/uso

Marca solo un óvalo.

- Nunca
- A veces
- Seguido
- Casi siempre
- Siempre
- No quiero contestar

20. Me cuesta separar mi vida personal de mi vida con los animales de laboratorio

Marca solo un óvalo.

- Nunca
- A veces
- Seguido
- Casi siempre
- Siempre
- No quiero contestar

21. No soy una persona productiva en el trabajo porque me cuesta conciliar el sueño, por el sufrimiento que viven los animales de laboratorio

Marca solo un óvalo.

- Nunca
- A veces
- Seguido
- Casi siempre
- Siempre
- No quiero contestar

22. Creo que me está afectando el sufrimiento que experimentan los animales que cuido/uso

Marca solo un óvalo.

- Nunca
- A veces
- Seguido
- Casi siempre
- Siempre
- No quiero contestar

23. Me siento preso por mi trabajo con animales de laboratorio

Marca solo un óvalo.

- Nunca
- A veces
- Seguido
- Casi siempre
- Siempre
- No quiero contestar

24. A raíz de mi trabajo de cuidar animales, muchas veces me siento al límite de mis emociones

Marca solo un óvalo.

- Nunca
- A veces
- Seguido
- Casi siempre
- Siempre
- No quiero contestar

25. Me gusta mi trabajo con animales de laboratorio

Marca solo un óvalo.

- Nunca
- A veces
- Seguido
- Casi siempre
- Siempre
- No quiero contestar

26. Me siento deprimido por el sufrimiento que experimentan los animales de laboratorio que cuido/uso

Marca solo un óvalo.

- Nunca
- A veces
- Seguido
- Casi siempre
- Siempre
- No quiero contestar

27. Siento como si estuviera experimentando el trauma que sufren los animales de laboratorio que cuido/uso

Marca solo un óvalo.

- Nunca
- A veces
- Seguido
- Casi siempre
- Siempre
- No quiero contestar

28. Tengo creencias (religiosas, eticas) que me sustentan

Marca solo un óvalo.

- Nunca
- A veces
- Seguido
- Casi siempre
- No quiero contestar

29. Estoy satisfecho con la forma en que puede manejar las técnicas de cuidado de animales de laboratorio y/o los protocolos experimentales

Marca solo un óvalo.

- Nunca
- A veces
- Seguido
- Casi siempre
- Siempre
- No quiero contestar

30. Soy la persona que siempre quise ser

Marca solo un óvalo.

- Nunca
- A veces
- Seguido
- Casi siempre
- Siempre
- No quiero contestar

31. Mi trabajo me hace sentir satisfecho

Marca solo un óvalo.

- Nunca
- A veces
- Seguido
- Casi siempre
- Siempre
- No quiero contestar

32. Me siento agotado por mi trabajo cuidando/usando animales de laboratorio

Marca solo un óvalo.

- Nunca
- A veces
- Seguido
- Casi siempre
- Siempre
- No quiero contestar

33. Tengo pensamientos y sentimientos positivos acerca de los animales que cuido/uso y como los cuido/uso

Marca solo un óvalo.

- Nunca
- A veces
- Seguido
- Casi siempre
- Siempre
- No quiero contestar

34. Me siento abrumado porque mi carga de trabajo parece ser interminable

Marca solo un óvalo.

- Nunca
- A veces
- Seguido
- Casi siempre
- Siempre
- No quiero contestar

35. Siento que puede hacer una diferencia en la vida de los animales a través de mi trabajo

Marca solo un óvalo.

- Nunca
- A veces
- Seguido
- Casi siempre
- Siempre
- No quiero contestar

36. Evito ciertas actividades o situaciones porque me recuerdan las experiencias desagradables que sufren los animales de laboratorio que cuido/uso

Marca solo un óvalo.

- Nunca
- A veces
- Seguido
- Casi siempre
- Siempre
- No quiero contestar

37. Me siento orgulloso de poder ayudar a los animales de laboratorio

Marca solo un óvalo.

- Nunca
- Raramente
- A veces
- Seguido
- Casi siempre
- Siempre
- No quiero contestar

38. Como resultado de mi trabajo como cuidador/investigador, tengo pensamientos intrusivos y desagradables

Marca solo un óvalo.

- Nunca
- A veces
- Seguido
- Casi siempre
- Siempre
- No quiero contestar

39. Siento que el sistema científico no se puede cambiar

Marca solo un óvalo.

- Nunca
- A veces
- Seguido
- Casi siempre
- Siempre
- No quiero contestar

40. Considero que soy muy bueno en mi trabajo

Marca solo un óvalo.

- Nunca
- A veces
- Seguido
- Casi siempre
- Siempre
- No quiero contestar

41. Me cuesta recordar detalles de mi trabajo que involucran el sufrimiento de los animales

Marca solo un óvalo.

- Nunca
- A veces
- Seguido
- Casi siempre
- Siempre
- No quiero contestar

42. Soy una persona muy amable en mi vida, en general

Marca solo un óvalo.

- Nunca
- A veces
- Seguido
- Casi siempre
- Siempre
- No quiero contestar

43. Estoy feliz de haber elegido este trabajo

Marca solo un óvalo.

- Nunca
- A veces
- Seguido
- Casi siempre
- Siempre
- No quiero contestar

Eutanasia

44. Alguna vez ha eutanasiado animales de laboratorio?

Marca solo un óvalo.

- Sí
- No
- No quiero contestar

45. Con que frecuencia eutanasia animales de laboratorio?

Marca solo un óvalo.

- Menos de una vez al mes
- Mensualmente
- Semanalmente
- Diariamente
- Nunca
- No quiero contestar

46. Cuál de los siguientes agentes eutanásicos ha utilizado?

Selecciona todos los que correspondan.

- [ ] Inyectable (por ejemplo anestésicos)
- [ ] Cámara de dióxido de carbono
- [ ] Dislocación cervical
- [ ] Decapitación
- [ ] Bala cautiva penetrante
- [ ] Martillo neumático
- [ ] Hipotermia/ congelamiento rápido
- [ ] Ninguno
- [ ] No quiero contestar
- [ ] Otro: ______________________

47. Puedo decidir si soy yo quien practica la eutanasia en los animales que cuido

Marca solo un óvalo.

- Nunca
- Algunas veces
- Siempre
- No aplica
- No quiero contestar

Apoyo social

48. Con qué frecuencia habla con otras personas sobre su trabajo con animales de laboratorio?

Marca solo un óvalo.

- Nunca
- A veces
- La mitad del tiempo
- Casi siempre
- Siempre
- No quiero contestar

49. Con qué frecuencia siente que puede contar con alguien en su lugar de trabajo, cuando está estresado en relación a su trabajo con animales de laboratorio?

Marca solo un óvalo.

- Nunca
- A veces
- La mitad del tiempo
- Casi siempre
- Siempre
- No quiero contestar

50. Se presenta ante la sociedad como una persona que trabaja con animales de laboratorio

Marca solo un óvalo.

- Nunca
- A veces
- La mitad del tiempo
- Casi siempre
- Siempre
- No quiero contestar

Comportamiento general

Por favor indique cuánto está en desacuerdo o acuerdo con los siguientes enunciados:

51. A menudo observo a los animales con los que trabajo

Marca solo un óvalo.

- Totalmente en desacuerdo
- En desacuerdo
- Algo de acuerdo
- De acuerdo
- Totalmente de acuerdo
- No quiero contestar

52. A menudo acaricio a los animales con los que trabajo

Marca solo un óvalo.

- Totalmente en desacuerdo
- En desacuerdo
- Algo de acuerdo
- De acuerdo
- Totalmente de acuerdo
- No quiero contestar

53. A menudo hablo con los animales con los que trabajo

Marca solo un óvalo.

- Totalmente en desacuerdo
- En desacuerdo
- Algo de acuerdo
- De acuerdo
- Totalmente de acuerdo
- No quiero contestar

54. A menudo le pongo nombre a los animales con los que trabajo

Marca solo un óvalo.

- Totalmente en desacuerdo
- En desacuerdo
- Algo de acuerdo
- De acuerdo
- Totalmente de acuerdo
- No quiero contestar

Cierre

55. Estaría dispuesto/a a participar de la entrevista virtual?

Marca solo un óvalo.

- Sí
- No
- No quiero contestar

Si desea participar de la encuesta, por favor envíe un mail a marcoppido.gisela@gmail.com y nos pondremos en contacto con usted

Este contenido no ha sido creado ni aprobado por Google.
